# A Functional Outcome on Different Surgical Treatment Modalities of Bimalleolar Fractures in Adults

**DOI:** 10.7759/cureus.104639

**Published:** 2026-03-03

**Authors:** Gokul S, Vivek Kandhasamy, Vijayakumar C S, Naveen Baskar S, Sriram M

**Affiliations:** 1 Department of Orthopaedics, inayaka Mission’s Kirupananda Variyar Medical College and Hospital, Vinayaka Mission’s Research Foundation (Deemed to be University), Salem, IND

**Keywords:** ankle fracture fixation, bimalleolar fractures, functional outcome, olerud-molander score, orif, syndesmotic stability

## Abstract

Objective: This study aimed to evaluate and compare the functional outcomes of adult patients with displaced closed bimalleolar ankle fractures treated with open reduction and internal fixation (ORIF) using different fixation modalities. The choice of implant and fixation technique was determined by fracture morphology, bone quality, and intraoperative stability assessment rather than age-specific criteria. The study further assessed whether functional outcomes differed across these modality-based treatment approaches.

Methodology: A prospective observational study was conducted between September 2023 and June 2025. It included 30 adult patients (aged 18-70 years) with displaced closed bimalleolar ankle fractures treated surgically using various fixation techniques, including cancellous compression (CC) screws, plates and screws, and tension band wiring (TBW). Postoperative outcomes assessed were fracture union time, range of motion (ROM), and functional status using the Olerud and Molander Ankle Score (OMAS). Statistical analyses were performed to evaluate associations between fixation methods and functional outcomes.

Results: Most patients were aged 21-40 years (n=16, 54%), and 18 (60%) were male. Road traffic accidents were the most common mechanism of injury (n=16, 53%), followed by falls from height (n=9, 30%) and twisting injuries (n=5, 17%) (χ²=6.18, df=2, p=0.045). Supination-external rotation and pronation-external rotation fractures were the most frequent patterns (n=9, 30% each). Fracture union was achieved in all patients. Functional outcomes were favorable: 26 patients (87%) demonstrated “good” objective outcomes, and 14 (47%) reported “excellent” subjective outcomes. No significant differences were observed in age distribution or functional outcomes among the fixation methods (p=0.34 and p=0.457, respectively). However, fracture type showed a significant association with age (F=12.13, p=0.0001).

Conclusion: Anatomical reduction with stable fixation using appropriate implants results in optimal restoration of ankle function following displaced bimalleolar fractures. Functional outcomes were comparable across fixation techniques when principles such as early surgical intervention, syndesmotic stability, and accurate joint alignment were maintained. Early fixation and precise anatomical reconstruction remain critical determinants of successful recovery.

## Introduction

Ankle fractures are a common lower limb injury, accounting for approximately 4-9% of all fractures and representing a substantial proportion of lower extremity fractures treated in clinical practice [1]. These injuries exhibit a bimodal age distribution, with higher rates in younger men and older women. Men show a higher incidence before age 50, whereas women predominate thereafter [2]. Common contributing risk factors include alcohol consumption and slippery surfaces, each responsible for nearly one-third of cases [2].

The ankle joint is a ginglymoid joint formed by the distal tibia with its medial malleolus, the lateral malleolus of the fibula, and the inferior transverse ligament, creating a confined cavity that articulates with the talus [3]. Joint stability is maintained by the medial collateral (deltoid) and lateral collateral ligaments, while the anteroinferior tibiofibular ligament (AITFL) forms part of the syndesmotic fibrous joint [3]. The blood supply of the ankle joint is derived from branches of the popliteal and peroneal arteries, and its innervation is provided by the deep peroneal nerve, medial cutaneous nerve of the leg, lateral sural cutaneous nerve, and posterior tibial nerve [4].

Twisting injuries and falls are the most common mechanisms of ankle fractures, frequently occurring during physical activity [2,5]. Comorbid conditions such as diabetes and obesity, particularly in middle-aged and elderly individuals, further increase the risk of fracture [5]. Most ankle fractures involve a combination of osseous and ligamentous injuries, with the fracture pattern determined by the direction and magnitude of the applied force [3].

Potts' early descriptive classification divided ankle fractures into unimalleolar, bimalleolar, and trimalleolar types [6]. Currently, the most widely used classification systems are the Danis-Weber and Lauge-Hansen classifications [7,8]. The Danis-Weber system categorizes fractures according to the level of the fibular fracture relative to the syndesmosis and assists in evaluating ankle stability [7]. In contrast, the Lauge-Hansen classification describes injury patterns based on the position of the foot and the deforming force at the time of injury. The major Lauge-Hansen patterns include supination-adduction (SAD), supination-external rotation (SER), pronation-abduction (PAB), and pronation-external rotation (PER) [8].

The primary goal of ankle fracture management is the restoration of anatomical alignment and joint stability [5]. While conservative treatment, including closed reduction, may be appropriate for selected stable fractures, open reduction and internal fixation (ORIF) remains the standard of care for unstable injuries, particularly bimalleolar fractures [5,9]. Functional outcomes following ORIF are commonly assessed using validated scoring systems such as the Olerud and Molander Ankle Score (OMAS), which evaluates pain and functional limitations after fixation [10,11]. Advances in fixation techniques, including precise anatomical reduction, syndesmotic stabilization, and early mobilization, have led to significant improvements in postoperative outcomes [9].

This study aimed to evaluate and compare the functional outcomes of adult patients with displaced closed bimalleolar ankle fractures treated with ORIF using different fixation modalities. The choice of implant and fixation technique was determined by fracture morphology, bone quality, and intraoperative stability assessment rather than age-specific criteria. The primary objective was to assess postoperative ankle range of motion (ROM) following fixation of both malleoli, as ROM restoration is essential for optimal functional recovery. Secondary objectives included determining the time to fracture union and its correlation with ROM and radiological outcomes, evaluating overall functional recovery using the OMAS, and assessing postoperative pain and related complications, all of which are clinically relevant indicators of surgical success and long-term ankle stability.

## Materials and methods

Study design and setting

This prospective observational study was conducted in the Department of Orthopedics at Vinayaka Mission's Kirupananda Variyar Medical College and Hospital, Salem, Tamil Nadu, India, from September 2023 to June 2025. Ethical approval was obtained from the Institutional Ethics Committee, and written informed consent was secured from all participants before enrollment.

Study population and sample size

The study included 30 adult patients aged 18-70 years with displaced closed bimalleolar ankle fractures who underwent surgical treatment during the study period. The sample size was calculated using the standard formula for estimating a single population mean: 

\[ n = \frac{Z^2 \times \sigma^2}{d^2} \]

where n represents the required sample size, Z is the standard normal deviate at a 95% CI (1.96), σ is the estimated SD derived from a previous comparable study [12], and d denotes the allowable error (precision). Based on the calculated sample size and feasibility within the study duration, a total of 30 patients were enrolled.

Inclusion and exclusion criteria

Adult patients aged 18-70 years with displaced closed bimalleolar ankle fractures who were medically fit for surgery and willing to participate were included in the study. Exclusion criteria included undisplaced fractures, open fractures, pediatric fractures, epiphyseal plate injuries, pathological fractures, delayed presentations with established malunion or nonunion, patients medically unfit for surgery, and those unwilling or unable to comply with follow-up requirements.

Preoperative assessment

All patients underwent a detailed clinical examination and radiological evaluation using standard anteroposterior, lateral, and mortise view radiographs of the ankle. Fractures were classified according to the Lauge-Hansen classification system, originally described by Lauge-Hansen [13]. This classification is a freely available, non-proprietary system and does not require permission or licensing for clinical or research use. Computed tomography (CT) scanning was not routinely performed for preoperative planning, as the study included closed displaced bimalleolar fractures in which standard radiographs provided sufficient information regarding fracture configuration, displacement, and ankle mortise alignment. CT imaging was reserved for selected cases with suspected posterior malleolar extension, complex comminution, or inconclusive radiographic findings. However, none of the enrolled patients required CT evaluation based on initial radiographic assessment. Demographic details, mechanism of injury, fracture pattern, side of involvement, and associated comorbidities were systematically recorded.

Surgical technique

ORIF was performed under spinal or regional anesthesia in accordance with AO principles (©2018 AO Foundation-AO/OTA fracture and dislocation classification compendium) [14], which is a freely available, open-access tool that does not require special permission for clinical or research use. All procedures were carried out by three experienced orthopedic surgeons. Fixation methods were selected based on fracture morphology and included corticocancellous screws, plate-and-screw constructs, tension band wiring (TBW), and K-wire fixation. Intraoperative assessment of syndesmotic stability was performed using the hook (Cotton) test [15], and syndesmotic screw fixation was undertaken when instability was identified. In all cases, emphasis was placed on achieving anatomical reduction, restoring fibular length, and maintaining congruity of the ankle mortise.

Postoperative management and follow-up

Postoperatively, patients were immobilized in a below-knee posterior slab for two weeks. Wound inspection and suture removal were performed between 12 and 14 days, after which ankle range-of-motion exercises were initiated. Partial weight-bearing was commenced following radiological evidence of early union, and full weight-bearing was permitted after confirmation of clinical and radiographic union. Follow-up assessments were conducted at six weeks, three months, six months, and one year postoperatively.

Outcome measures

Functional outcomes were assessed using the OMAS (©2006 Lippincott Williams & Wilkins, Inc) [16], a validated patient-reported outcome measure that evaluates pain, stiffness, swelling, stair climbing, running, jumping, squatting, need for supports, and activities of daily living. As OMAS is a subjective functional score completed by the patient, it was used solely for subjective assessment of postoperative recovery.

Objective evaluation included clinical measurement of ROM using a goniometer and radiological assessment of fracture union and ankle mortise alignment on follow-up radiographs. Radiological union was defined as the presence of bridging callus across the fracture site, with the absence of tenderness at the fracture site. Postoperative complications, including wound infection, wound dehiscence, and implant-related issues, were documented at each follow-up visit.

Statistical analysis

Data were entered into Microsoft Excel and analyzed using Statistical Package for the Social Sciences (SPSS) version 20.0 (IBM Corp., Armonk, NY, USA). Descriptive statistics were reported as mean±SD for continuous variables and as frequencies with percentages for categorical variables. Comparisons between categorical variables were performed using the chi-square test. Continuous variables, including mean age across different fixation modalities and fracture classifications, were compared using one-way analysis of variance (ANOVA). When ANOVA demonstrated statistical significance, an appropriate post hoc analysis using Tukey's honestly significant difference (HSD) test was performed to identify intergroup differences. A p-value of <0.05 was considered statistically significant.

## Results

The majority of patients belonged to the 21-40-year age group (16 patients, 54%), with males comprising 18 (60%) of the study population. Road traffic accidents were the most common mechanism of injury, accounting for 16 (53%) cases, followed by falls from height in nine (30%) patients and self-falls or twisting injuries in five (17%) patients. This distribution showed a statistically significant difference (χ²=6.18, df=2, p=0.045). SER and PER injury patterns were the most frequently observed, each occurring in nine (30%) patients. Regarding fixation methods, corticocancellous screw fixation was used in 13 (43%) patients, plate-and-screw constructs in 12 (40%), and TBW in five (17%). No statistically significant differences were observed in the distribution of age, sex, injury patterns, or implant types among the groups (p>0.05) (Table 1).

**Table 1:** Comparison of mean age across different modes of fixation and fracture classifications *Significant p-value. A p-value less than 0.05 was considered significant. TBW, tension band wiring; SAD, supination adduction; SER, supination external rotation; PAB, pronation abduction; PER, pronation external rotation

Variables	Total number, N (%)	Chi-sqaure	df	p-vaule
Age in years		7.21	5	0.206
11 to 20	2 (6%)			
21 to 30	8 (27%)			
31 to 40	8 (27%)			
41 to 50	5 (17%)			
51 to 60	5 (17%)			
61 to 70	2 (6%)			
Sex		1.20	1	0.273
Male	18 (60%)			
Female	12 (40%)			
Cause of injury		6.18	2	0.045*
Road traffic accident	16 (53%)			
Fall from height	9 (30%)			
Self fall, twisting	5 (17%)			
Injury patterns		2.25	3	0.519
SAD	8 (27%)			
SER	9 (30%)			
PAB	4 (13%)			
PER	9 (30%)			
Implant used		3.79	2	0.150
Corticocancellous screw	13 (43%)			
Plate and screws	12 (40%)			
TBW	5 (17%)			

Functional outcomes assessed using both objective and subjective scoring systems demonstrated favorable results in the majority of patients. Based on objective scoring, 26 (87%) patients were graded as "good," three (10%) as "fair," one (3%) as "poor," and none as "excellent." In contrast, subjective scoring showed 14 (47%) patients rated as "excellent," 12 (40%) as "good," three (10%) as "fair," and one (3%) as "poor." Overall, these findings indicate satisfactory to excellent recovery in most patients, with subjective assessments reflecting slightly better perceived outcomes than objective evaluations (Table 2).

**Table 2:** Functional outcomes according to objective and subjective scoring systems

Categories	Objective scoring, N (%)	Subjective scoring, N (%)
Excellent	0 (0%)	14 (47%)
Good	26 (87%)	12 (40%)
Fair	3 (10%)	3 (10%)
Poor	1 (3%)	1 (3%)

Analysis of patient age across different fixation modalities revealed no statistically significant difference (F=1.15,p=0.34), suggesting that age did not influence the choice of surgical fixation. However, fracture classification demonstrated a significant association with age (F=12.13,p=0.0001). Patients with SER 4 fractures were generally younger, those with SAD 2 fractures were older, and patients with PER 3 fractures exhibited an intermediate age distribution. These findings suggest that patient age may influence the type of ankle fracture sustained, but does not determine the fixation method selected (Table 3).

**Table 3:** Comparison of mean age across different modes of fixation and fracture classifications CC, cancellous compression; TBW, tension band wiring; SER, supination-external rotation; PER, pronation-external rotation; SAD, supination-adduction; LM, lateral malleolus; MM, medial malleolus

Variables	Mean age (years)	One-way ANOVA	p-value
Mode of fixation		1.15	0.34
CC screw/LM plate and screws	35		
TBW/LM plate and screws	43		
MM plate and screws/LM plate and screws	48		
Plate and screws/LM K-wire	39		
Fracture classification (Lauge-Hansen)		12.13	0.0001
SER 4	36		
PER 3	47		
SAD 2	55		

**Figure 1:**
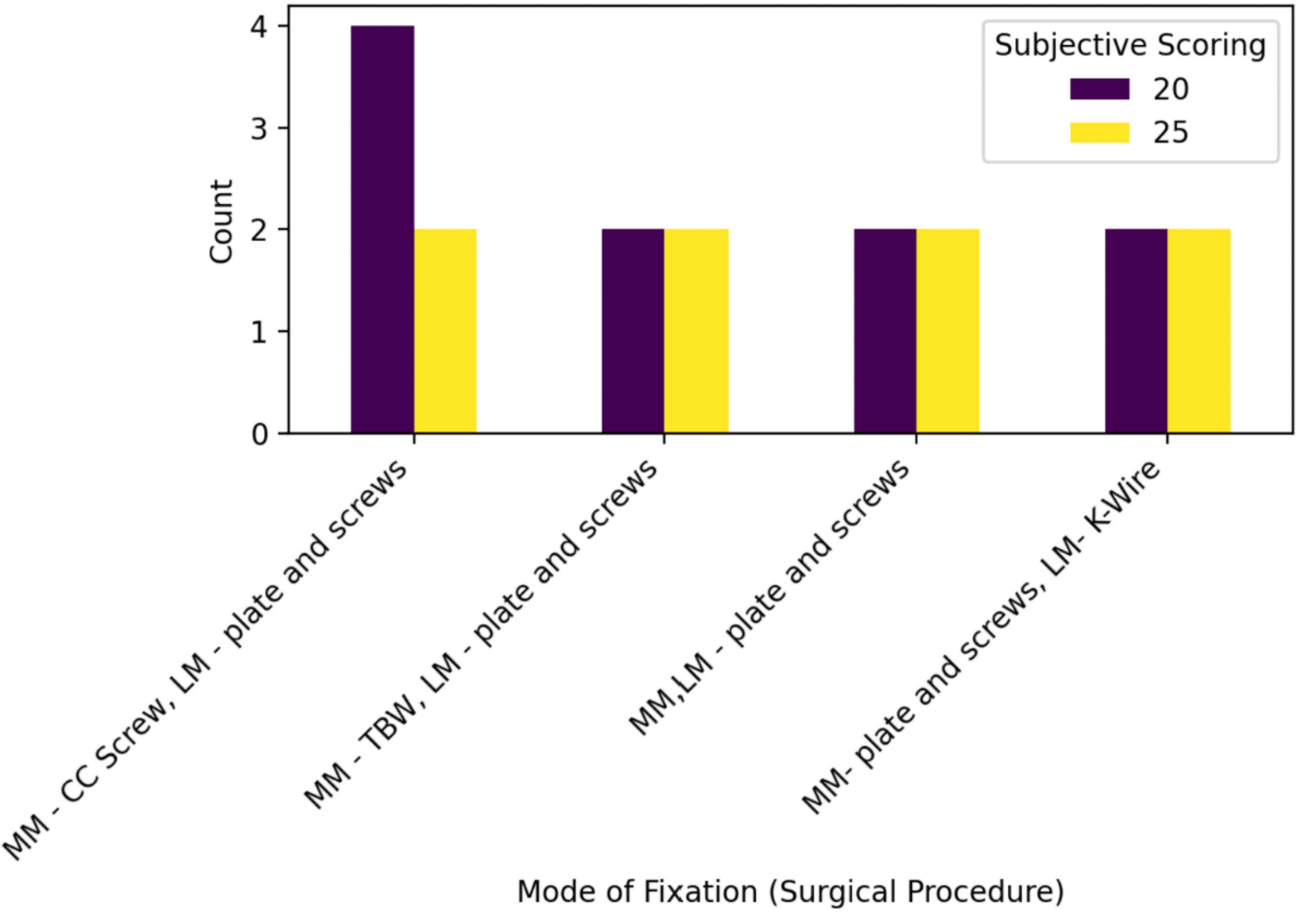
Distribution of subjective scores by mode of fixation CC, cancellous compression; TBW, tension band wiring; LM, lateral malleolus; MM, medial malleolus

Subjective outcome analysis showed a narrow distribution of scores across fixation modalities, with scores of 20 observed in two to four patients and scores of 25 in two patients per group. No fixation technique demonstrated numerical superiority in subjective scoring, indicating comparable patient-reported outcomes when fixation methods were chosen based on fracture morphology (Figure 1).

**Figure 2:**
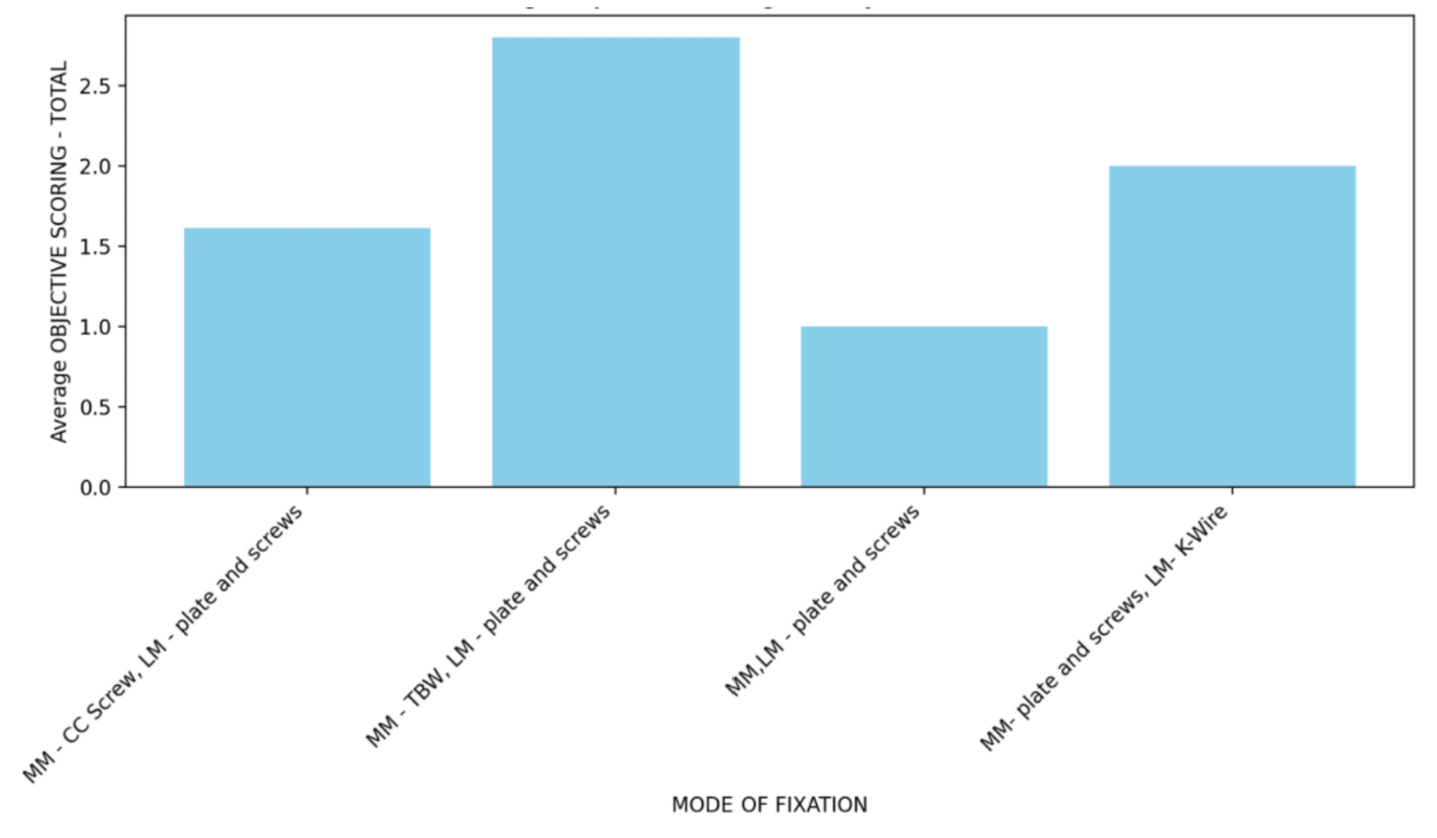
Average objective score by mode of fixation CC, cancellous compression; TBW, tension band wiring; LM, lateral malleolus; MM, medial malleolus

As illustrated in Figure 2, the highest mean objective score was observed in patients treated with medial malleolar TBW combined with lateral malleolar plating (mean≈2.8), followed by medial malleolar plate-and-screw fixation with lateral malleolar K-wire fixation (mean≈2.0). Lower mean scores were noted in patients treated with medial malleolar cancellous compression (CC) screw and lateral malleolar plate fixation (mean≈1.6) and bimalleolar plate-and-screw fixation (mean≈1.0). Despite these numerical differences, the variation in objective scores among fixation methods was not statistically significant (p=0.4568), indicating comparable objective functional outcomes when fixation techniques were appropriately selected according to fracture characteristics.

## Discussion

In this study, closed bimalleolar ankle fractures were most frequently observed in middle-aged patients, particularly those in their third and fourth decades. Among the 30 cases analyzed, males predominated (60%) compared to females (40%), and the right ankle was affected in 60% of cases. Road traffic accidents were the leading cause of injury (53%), followed by falls from height (30%) and self-falls or twisting injuries (17%).

SER and PER injuries were the most common fracture patterns, with nine cases each, followed by SAD in eight patients and PAB in four patients. Stress radiographs are a valuable tool for assessing ankle joint stability and profound deltoid ligament integrity. Gravity stress radiographs are more sensitive and better tolerated than manual testing; however, Weber [11] cautioned that stress radiographs may overestimate instability. Nevertheless, they remain useful in distinguishing SER type II from SER type IV injuries. Unstable fractures, especially SER type IV, require restoration of ligamentous integrity and syndesmotic stability [17].

Among patients with SER injuries, seven out of nine achieved good to excellent functional outcomes, highlighting the importance of early intervention, as promptly treated dislocations had better results. All SAD-type injuries also resulted in good to excellent outcomes. Small fibular fragments were managed with K-wire or screw fixation, and an anteromedial approach, as described by Hamilton et al. [17], was preferred over the traditional hockey-stick incision.

In PAB injuries, medial malleolar fixation followed by fibular plating was performed to restore anatomical alignment and reduce the risk of nonunion, in line with current principles of stable fixation for complex bimalleolar fractures [17,18]. For PER injuries, preservation of fibular length, correct rotation, and restoration of syndesmotic stability are critical to maintaining ankle mortise congruity. Using fibular plating combined with syndesmotic screw fixation, all seven PER cases achieved good to excellent outcomes [19,20].

Fracture displacement depends on talar position and the integrity of the deep deltoid ligament. Fixation of the malleolar fragment alone does not ensure stability; direct repair of the deep deltoid ligament is required when disrupted. Stable fractures do not displace under axial loading [17]. Accordingly, management should be guided by fracture stability, while prognosis is influenced by the energy of injury. Although the Lauge-Hansen classification provides detailed mechanisms of injury, it does not account for syndesmotic injuries [8].

Patients treated with fibular K-wires demonstrated inferior outcomes compared to those receiving contoured reconstruction plates or one-third tubular plates, likely due to improved control of fibular valgus and enhanced fracture stability with plate fixation.

Intraoperatively, syndesmotic stability was assessed using the Cotton (hook) test [15]. The AO Foundation [14] emphasizes the importance of these tests for detecting syndesmotic disruption and ankle instability. Boden et al. [21] suggested that syndesmotic fixation may be unnecessary if rigid medial fixation is achieved; however, it is recommended for fibular fractures extending more than 4.5 cm above the ankle in the absence of stable medial fixation.

When combined, the excellent and good subjective outcomes in the present study accounted for 87% of cases (47% excellent and 40% good), which is slightly higher than the 84% excellent-to-good rate reported by Hafiz et al. [22]. Similarly, objective good outcomes were observed in 87% of our patients, compared to 78.8% reported in their series. The proportion of poor outcomes in our study (3%) was also marginally lower than the 4.2% reported by Hafiz et al. These direct comparisons suggest that the functional outcomes achieved in the present study are consistent with, and in some aspects slightly favorable compared to, previously published literature.

Postoperative complications included wound infection in three patients and wound dehiscence in one patient, with superficial infection and skin necrosis being the most common. Use of a low-profile 3.5-mm plate-and-screw system was associated with reduced skin necrosis. Kang et al. [23] reported a 2.2% infection rate in their bimalleolar fracture series. They recommended a direct incision to the bone without undermining the skin or subcutaneous tissue to minimize soft-tissue complications. The findings of the present study highlight important clinical implications. Functional outcomes were comparable across different fixation techniques, suggesting that implant selection should be guided primarily by fracture morphology and intraoperative stability rather than patient age alone. Careful anatomical reduction, restoration of syndesmotic stability, and adherence to soft-tissue handling principles appear to be more critical determinants of successful recovery than the specific implant choice.

Limitations

This study has several limitations that should be considered when interpreting the results. First, the relatively small sample size and single-center design limit the generalizability of the findings to a broader population. Second, the observational nature of the study and the absence of a control or comparison group preclude definitive conclusions regarding the superiority of one fixation method over another. Third, although functional outcomes were assessed using the validated OMAS, patient-reported measures are inherently subjective and may be influenced by individual expectations and adherence to rehabilitation protocols. Fourth, while the follow-up duration was adequate for assessing short- to mid-term outcomes, it may not have been sufficient to evaluate long-term complications such as post-traumatic ankle arthritis. Finally, variations in fracture patterns and fixation techniques, influenced by fracture morphology and surgeon preference, may have introduced treatment heterogeneity that could affect outcome comparisons. In addition, as the procedures were performed by three experienced orthopedic surgeons, inter-surgeon variability in surgical technique and intraoperative decision-making may represent a potential source of heterogeneity. However, all surgeries were performed according to standard AO principles and uniform postoperative rehabilitation protocols, which may have minimized variability in overall outcomes. Despite these limitations, this study provides valuable insights into the functional outcomes of surgically treated displaced bimalleolar ankle fractures.

## Conclusions

In the present study, SER and PER injuries were the most common patterns of displaced bimalleolar ankle fractures. Overall, satisfactory functional outcomes were observed following ORIF across the various fixation modalities. Although no statistically significant differences in functional outcomes were identified between fixation techniques, the study was not designed to establish equivalence between methods. Favorable outcomes were observed in patients in whom anatomical alignment and syndesmotic stability were restored, consistent with established principles of ankle fracture management. However, given the observational design and relatively small sample size, these findings should be interpreted with caution. Further studies with larger sample sizes and controlled designs are required to more definitively evaluate comparative effectiveness between fixation methods and the influence of surgical variables on long-term functional outcomes.
